# Cellular senescence in lymphoid organs and immunosenescence

**DOI:** 10.18632/aging.203405

**Published:** 2021-08-12

**Authors:** Vivekananda Budamagunta, Thomas C. Foster, Daohong Zhou

**Affiliations:** 1 Genetics and Genomics Graduate Program, Genetics Institute, College of Medicine, University of Florida, Gainesville, FL 32610, USA; 2 Department of Neuroscience, McKnight Brain Institute, College of Medicine, University of Florida, Gainesville, FL 32610, USA; 3 Department of Pharmacodynamics, College of Pharmacy, University of Florida, Gainesville, FL 32610, USA

**Keywords:** cellular senescence, immunosenescence, immune senescence, senescence associated secretory phenotype (SASP), thymus

## Abstract

Immunosenescence is a multi-faceted phenomenon at the root of age-associated immune dysfunction. It can lead to an array of pathological conditions, including but not limited to a decreased capability to surveil and clear senescent cells (SnCs) and cancerous cells, an increased autoimmune responses leading to tissue damage, a reduced ability to tackle pathogens, and a decreased competence to illicit a robust response to vaccination. Cellular senescence is a phenomenon by which oncogene-activated, stressed or damaged cells undergo a stable cell cycle arrest. Failure to efficiently clear SnCs results in their accumulation in an organism as it ages. SnCs actively secrete a myriad of molecules, collectively called senescence-associated secretory phenotype (SASP), which are factors that cause dysfunction in the neighboring tissue. Though both cellular senescence and immunosenescence have been studied extensively and implicated in various pathologies, their relationship has not been greatly explored. In the wake of an ongoing pandemic (COVID-19) that disproportionately affects the elderly, immunosenescence as a function of age has become a topic of great importance. The goal of this review is to explore the role of cellular senescence in age-associated lymphoid organ dysfunction and immunosenescence, and provide a framework to explore therapies to rejuvenate the aged immune system.

## INTRODUCTION

### The aging population

Aging is the gradual process of organismal deterioration which is associated with a multitude of age-related disorders and diseases that make one wonder if aging itself is a disease that needs to be addressed [1]. A shadow is cast on the benefits of longevity if the elderly are faced with the possibility of a decline in their quality of life. The world currently has over 700 million people who are over the age of 65, a number that is projected to grow rapidly in the near future [2]. As advancing age is strongly correlated to decreased quality of life and increased risk of several age-related diseases [3], these demographics seem more dismal in prospering countries, with the USA and the UK having about 16–18% of their population over the age of 65 [4, 5]. With the life expectancy of most Western countries steadily increasing, majority of people are expected to spend at least 2 decades, or 25% of their life, over the age of 65, when they are prone to acquiring various age-related morbidities [6, 7]. The silver lining to this otherwise tragic situation is that results from recent studies indicate that the aging process and the pace of organismal deterioration is malleable and can be influenced greatly by physiological, genetic, dietary and pharmaceutical interventions [8–16].

### The aging immune system

The immune system is a complex network of cells and tissues working in coalition to maintain the health of an organism. It not only clears foreign pathogens, but also helps to maintain the integrity of the organism by clearing away dead or dysfunctional cells [17–22]. Due to the immune system’s complexity and intricacy, 7% of the genes from the human genome are allocated exclusively for its functioning and maintenance [23].

Like any other system, the immune system changes with age and experiences gradual deterioration. Improving our understanding of this phenomenon is of great significance because the medical and scientific advancements that have facilitated the unprecedented increase in average human lifespan have been unable to significantly increase the human healthspan [24]. Because of this, we have a rapidly increasing aging population in a world where there is a substantial risk of steep decline in quality of life with age.

Age-associated deterioration and dysfunction of the immune system leads to the establishment of an incompetent immune response against invading pathogens [25, 26]. This could partially provide an explanation for the age-dependent increase of mortality in patients suffering from infections like influenza [27], with people older than 65 accounting for more than 90% of the influenza-associated annual deaths [28]. Furthermore, the aged immune system elicits an inadequate response to vaccines, leaving the elderly susceptible to pathogens despite being vaccinated against them [29, 30]. This is especially poignant in the wake of an ongoing pandemic where the mortality rate is disproportionately high in the elderly [31].

Aging of the immune system is also one of the major factors that accelerates the deterioration of an organism, as its dysfunction not only fails to elicit a strong immune response against invading pathogens but also drives the accumulation of undesirable and malfunctioning cells [25, 32–36]. In some cases the aging immune system also develops an affinity for attacking self-antigens, leading to autoimmunity-associated disorders [37, 38].

In recent years, there have been many studies that have broadened our understanding of the aging immune system and immunosenescence (the gradual deterioration of the immune system with age) from the perspective of genetics, nutrition, physiology, and molecular biology [39–42]. Despite this assimilation of knowledge, a complete understanding of the dynamics of this process is lacking.

Within a systemic context, the age-related changes and adversities in any organ system arise from a complex crosstalk between different cells and processes of the body. By virtue of the way that research studies are designed and funded, many aspects of this complexity are often overlooked. In this review, we will discuss one such interaction, between cellular senescence and the immune system with a focus on the accumulation of SnCs in the lymphoid organs of the aging body, which is greatly understudied and underappreciated.

### Cellular senescence

Initially described in 1961, cellular senescence is the phenomenon by which cells cease to divide despite the availability of adequate growth factors [43]. It was later established that upon encountering certain types of stress and irreparable damage, cells tend to enter a stable cell cycle arrest [44]. From an evolutionary perspective, this is widely considered to be a protective mechanism to prevent the stressed and damaged cells from becoming deleterious to the body.

Like most things optimized by evolution, cellular senescence is not of much concern to the younger body capable of reproduction while the older body, past its reproductive prime, is adversely affected by it. The fitness benefits that cellular senescence provides to younger, reproductively active animals, such as preventing cancer [45], mitigating the progression of fibrosis [46–48] and promoting optimal wound healing [49], have helped the phenomenon survive the arduous tests of natural selection over the millennia. Unfortunately, in almost an antagonistically pleiotropic manner, accumulation of SnCs is very detrimental to the older body [50]. Specifically, SnCs secrete various factors classified together as senescence-associated secretory phenotype (SASP) which cause instability and dysfunction in their surrounding environment [51]. Both SnCs and SASP factors have been implicated in many of the age-related deteriorations, dysfunctions and diseases including but not limited to frailty, hypertrophy of tissue, stem-cell exhaustion, bystander effect mediated senescent cell accumulation, and cancer [51–63].

The interactions between SnCs and the immune system run in both directions, with the immune system surveilling and clearing the SnCs; while the SnCs frequently impede the function, and in some contexts, generation of immune cells. In young and healthy individuals, the immune system can rapidly clear SnCs after their induction, which prevents them from significantly accumulating and causing adverse effects [18, 64]. In older individuals, this turnover is slow and leads to the accumulation of SnCs [34]. Ovadya et al. demonstrated that accumulation of SnCs is accelerated upon impaired immune surveillance [32]. Since advancing age is associated with impairment in immune function [65], the decline in the turnover of SnCs with age can, at least partially, be attributed to this impediment. Despite multiple studies demonstrating various mechanisms via which SnCs could evade immune clearance [66, 67], the impact of aging on immune evasion of SnCs is not yet completely understood. Of note, SnCs have been shown to cause stem cell exhaustion and dysfunction [62, 68–72]. This is of great relevance and importance to the topic of immunosenescence because senescence, exhaustion and dysfunction of hematopoietic stem cells (HSCs), causes myeloid skewing and a decrease in the production of immune cells which may be one of the underlying causes of age-related immunosenescence.

**Figure 1:**
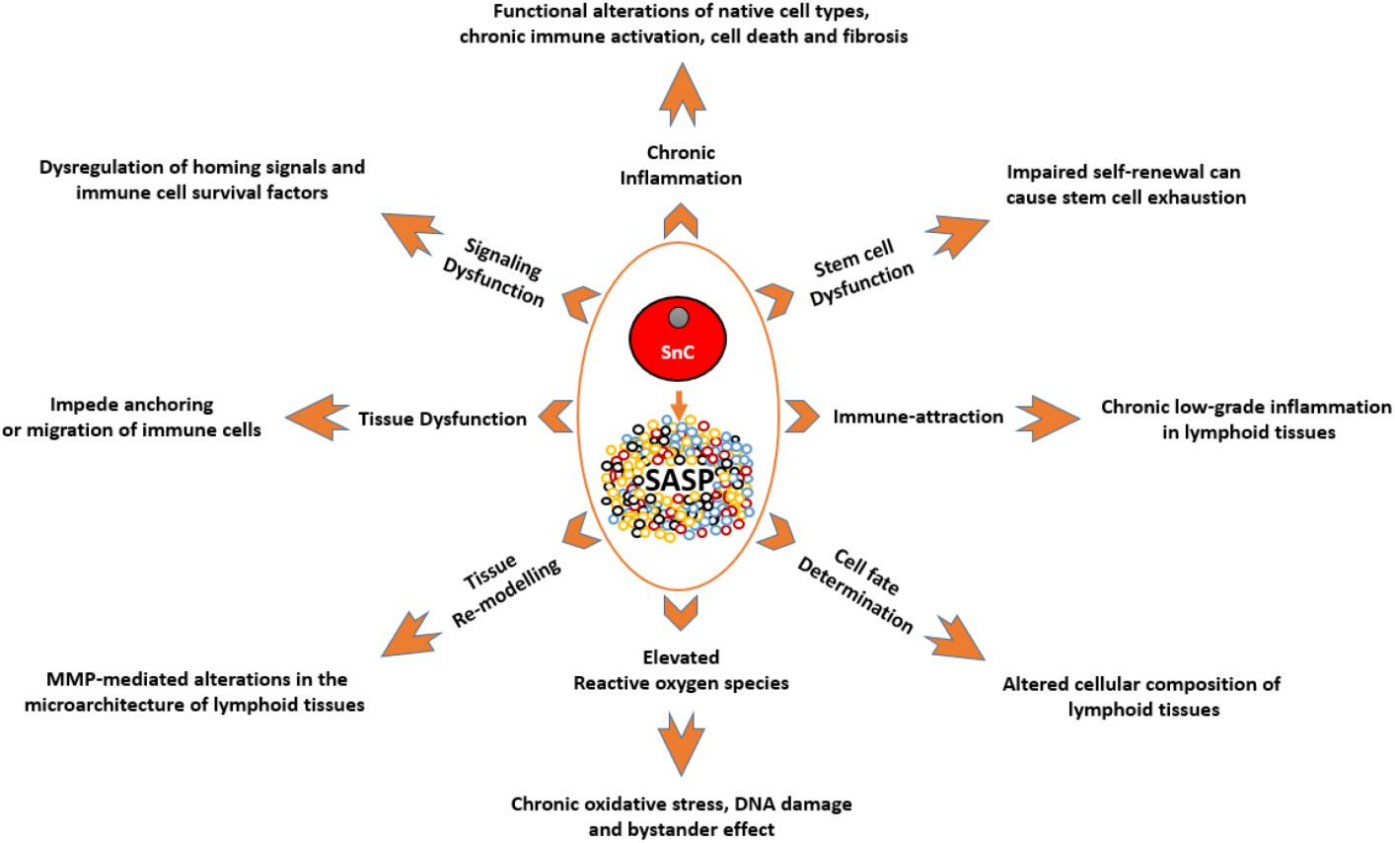
**A depiction of the known effects of SnCs and SASP on different cell types and tissues, and how they are relevant to the immune system.** SnCs possess altered morphology and surface markers and usually fail to perform the tasks of their non-senescence counterparts. This makes them the dysfunctional units of a tissue which can impede normal functions such as, immune cell priming and transmigration. MMPs produced by SnCs can modify the surrounding matrix and alter the microarchitecture of the lymphoid organs. As these organs are precisely organized into zones with specialized functions, such micro-architectural alterations can lead to dysfunction. SASP produced by SnCs can act as a chemoattractant to immune cells which can lead to unresolved chronic inflammation in tissues. SASP by itself can be inflammatory which can adversely impact neighboring cells. This chronic unresolved inflammation can lead to pathological conditions like fibrosis and neoplasia. SASP-mediated signaling and ROS-mediated oxidative stress can impair clonogenicity and functionality of HSCs, immune cells and other supporting cells of the immune system. SnCs and SASP can alter the expression profile of supporting cells leading to the dysregulation of homing signals required for proper localization of immune cells, and survival factors required for the endurance of certain immune cells. SnCs, by means of SASP, can influence the cell fate of differentiating cells and in some cases, cause the accumulation of adipocytes in the lymphoid organs. Abbreviations: SnC: Senescent cell; SASP: Senescence associated secretory phenotype; MMPs: Matrix metalloproteases; ROS: Reactive Oxygen Species; HSC: Hematopoietic stem cell.

With many more possible domains of interaction between cellular senescence and the immune system, as seen in (Figure 1), this review will discuss literature that states or suggests the presence of this interaction, with a focus on cellular senescence in the lymphoid organs, and raises questions that need to be answered to strengthen the foundation of the role of cellular senescence in immunosenescence.

## Cellular Senescence in the Organs of the Immune System

### Bone marrow

Bone marrow is a spongy tissue residing in the core of vertebrae, skull and long bones. It is the home of HSCs which give rise to most of the immune cells [73]. HSCs are self-renewing pluripotent cells that can generate the entire hematopoietic system.

**Figure 2:**
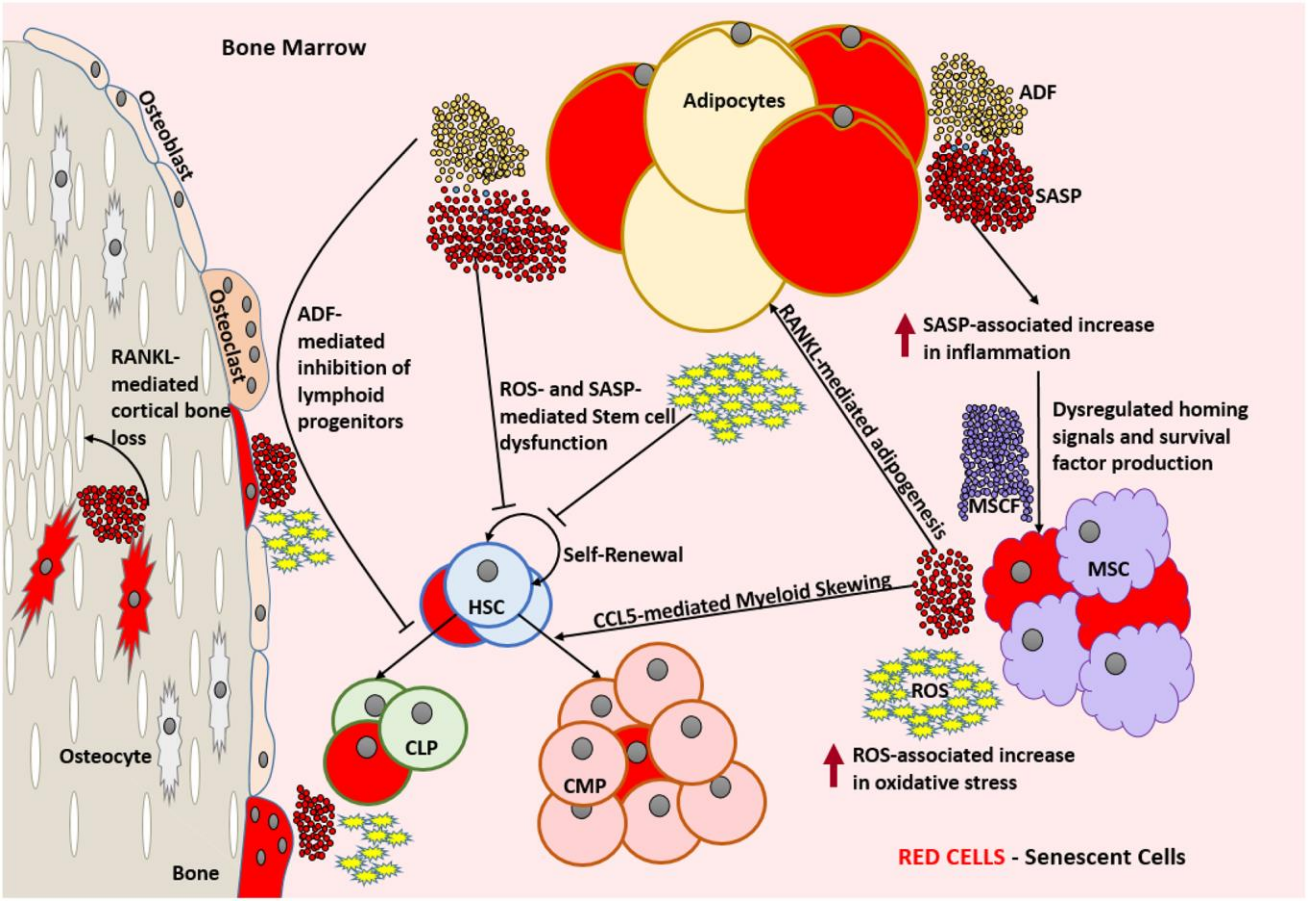
**Aged bone marrow microenvironment with accumulated SnCs is not conducive for its normal functionality.** SASP and ROS mediate dysfunction and DNA damage in HSCs, respectively and lead to a change in the HSC repertoire and exhaustion of the functional HSC reservoir. RANKL mediates the accumulation of adipocytes that produce ADFs. CCL5 and ADFs mediate the establishment of myeloid skewing in HSCs. SASP mediated inflammation can dysregulate the adequate production of homing signals and survival factors by the MSCs which can lead to the depletion of selective immune cell types. The increased ROS and SASP mediated inflammation causes damage to the surrounding cells and induces senescence by means of the bystander effect. SnCs such as osteocytes can produce SASP that is detrimental to the bone housing which encloses them. In the absence of rapid clearance of SnCs, this becomes a self-perpetuating cycle of dysfunction and damage causing severe immunosenescence. Abbreviations: SnC: Senescent cell; SASP: Senescence associated secretory phenotype; ROS: Reactive Oxygen Species; HSC: Hematopoietic stem cell; CLP: Common lymphoid progenitor; CMP: Common myeloid progenitor; MSC: Mesenchymal stem cell; MCSF: Mesenchymal stem cell derived factors; ADF: Adipocyte derived factors; CCL5: Chemokine Ligand 5; RANKL: Receptor activator of nuclear factor kappa-Β ligand.

With increasing age, the bone marrow microenvironment changes dramatically. With advancing age, HSC number increases, while their functionality, including self-renewal and clonogenicity declines. These changes are accompanied by myeloid skewing, elevated adipogenesis in the bone marrow, and alterations in the bone marrow niche [74–78]. Along with the prevalence of significantly more apoptotic cells, bone marrow cellularity (volume occupied by HSCs) decreases significantly with age reaching values lower than 40% [79]. A graphic depiction of the aged bone marrow microenvironment is illustrated in (Figure 2).

Myeloid skewing of HSCs with aging may be in part attributable to the aged bone marrow microenvironment, as even young HSCs develop a myeloid bias upon being transplanted into old mice [80, 81]. It has been suggested that chemokine ligand 5 (CCL5) is a major factor that drives myeloid skewing of HSCs with advancing age. Over expression of CCL5 causes a decrease in pro-lymphoid transcription factors and T-cell differentiation, while genetically knocking out CCL5 prevents myeloid skewing in mice [82]. Age-related accumulation of adipocytes in the bone marrow has been attributed to the increased expression of receptor activator of nuclear factor kappa-B ligand (RANKL) [83]. These bone marrow adipocytes in-turn produce an array of factors that have been shown to affect hematopoiesis and skew it towards myeloid lineage [84–88].

The accumulation of various p16^INK4a^ positive cells [89–91], SASP factor (like CCL5 and RANKL) generating cells [91–94] in aged bone marrow, along with increased number of cells harboring DNA damage and elevated ROS [95, 96], suggests that age-dependent bone marrow changes can be in part attributed to the accumulation of SnCs.

Based on data showing that the expression profile of adipocytes resembles the SASP profile of SnCs [97], it is likely that a great proportion of these adipocytes are senescent. This became evident after a study where clearance of SnCs in INK-ATTAC mice, a genetically altered model that clears cells expressing *p16^INK4a^*, showed a significant reduction in the number, size, and tissue volume of bone marrow adipocytes [98]. Other studies have also shown that, despite the structural and functional support provided by adipocytes, they adversely influence the hematopoietic environment [99, 100]. However, whether this is completely attributable to senescent adipocytes and their SASP is yet to be determined.

A recent study implicated the senescence of bone marrow-derived mesenchymal stem cells (BM-MSCs) in the age-associated dysfunction of HSCs, in humans. This study revealed that a significantly higher portion of senescent MSCs were seen in the bone marrow explants of the elderly when compared to their younger cohorts. This was established by showing increased accumulation of cells with DNA damage, elevated ROS and SASP expression. They also showed that the functionality and clonogenicity of young HSCs were impaired when exposed to factors generated by these MSCs [95]. The inflammatory environment, created by SASP of these SnCs, can alter the expression profile of normal MSCs to dysregulate the expression of factors necessary for lymphocyte survival [101–105].

Along with the cell-extrinsic causes for stem cell aging, older HSCs show an accumulation of senescence in association with increased DNA damage and telomere attrition, along with having an increased risk of undergoing an inflammatory cell death known as pyroptosis [68, 106]. Reactive oxygen species (ROS) produced by SnCs play a key role in the bystander effect [107]. ROS produced by SnCs in the bone marrow environment can cause DNA breaks in HSCs. This agrees with the finding that aged HSCs harbor more DNA damage compared to their younger counterparts [108]. As the DNA damage repair mechanism is not robust and quite error prone in the quiescent HSCs [109], the constant oxidative stress-induced DNA damage can progressively deplete and alter the functional HSC repertoire with increasing age [110].

Direct evidence for the adverse role of cellular senescence in modulating HSC function during aging was provided by demonstrating that knocking out *p16^INK4a^* conserved HSC functionality and stress tolerance with age [68]. A more recent study from our lab has shown that clearing SnCs rejuvenated the aging HSC repertoire by reducing myeloid skewing and improving clonogenicity significantly in mice [63].

### Thymus

The thymus is a primary lymphoid organ located behind the breastbone and above the heart, within which T-cells mature. In an evolutionarily conserved manner, most vertebrates experience an age-associated thymic involution, which is characterized by atrophy and the development of cavities. An age-dependent alteration in thymic cellularity can be seen, with most functional cells getting replaced by fibroblasts, fat cells and senescent cells [111–116].

**Figure 3:**
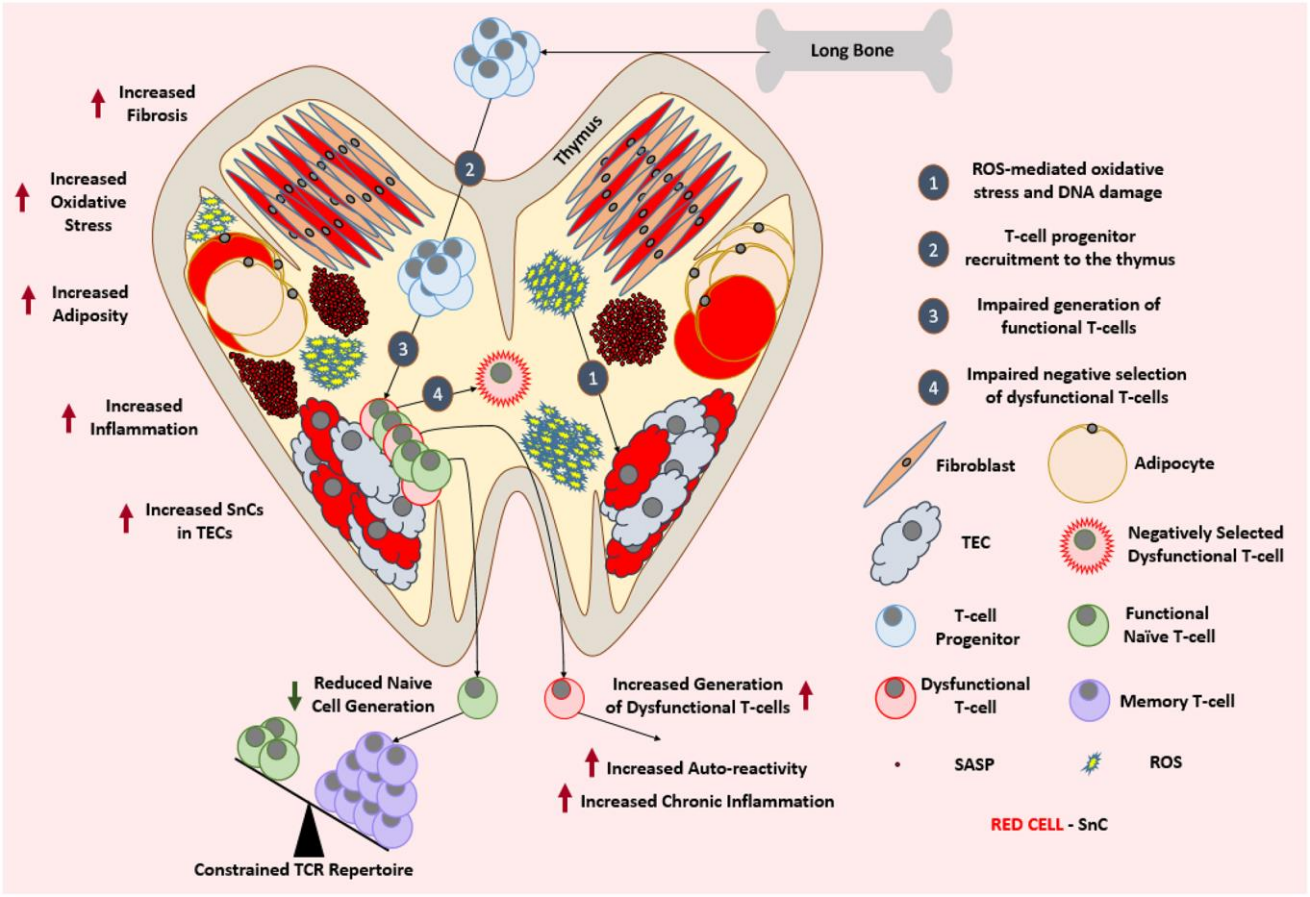
**Aged thymus is dysfunctional.** With advancing age, thymus loses its cellularity while accumulating adipocytes and fibroblasts. Aged thymus develops an inflammatory environment with high levels of oxidative stress. This is evident by the accumulation of senescent TECs with elevated markers of DNA damage and oxidative stress. Despite the adequate recruitment of T-cell progenitors, aged thymus generates inadequate number of naïve T-cells which leads to the age-associated depletion of TCR repertoire and ultimately a change in the immune cell landscape. Due to the impaired negative selection of dysfunctional T-cells, the aged thymus shows an increase in the output of dysfunctional and autoreactive T-cells leading to the establishment of low-grade chronic inflammation. Abbreviations: SnC: Senescent cell; SASP: Senescence associated secretory phenotype; ROS: Reactive Oxygen Species; TEC: Thymic epithelial cell; TCR: T-cell receptor.

Thymic atrophy is associated with the reduced turnover of new T-cells [117], a constricted T-cell receptor repertoire [118] and the production of higher autoreactive T-cells that could lead to autoimmunity [119]. As depicted in (Figure 3) these are characteristic features of immunosenescence that play an important role in age-associated impaired T-cell function [120].

Thymic epithelial cells (TECs) from adult human thymus stained positive for senescence- associated beta galactosidase (SA-βGal) and the thymic tissues from these adults also strongly stained positive for markers of oxidative DNA damage such as γH2AX and 8-oxoguanine [121]. A similar finding of high γH2AX staining was seen in the thymus of old mice, which was indicative of DNA damage and cellular senescence [111]. This also correlated with the increased inflammatory environment of the aged thymus seen in humans [122]. Despite the abundance of evidence suggesting accumulation of SnCs in atrophied thymus, whether cellular senescence plays a causal role in thymic involution needs to be further studied, as the accumulation of SnCs could be a consequence of thymic involution. But the possibility of a causal involvement of SnCs and their SASP seems likely because the administration of IL-6, a known SASP factor, has been shown to induce thymic atrophy [122]. In addition, increased oxidative stress and DNA damage in the stromal cells, especially TECs, has also been shown to accelerate thymic aging [123].

With the existing knowledge that TECs play a crucial role in the positive and negative selection of maturing T-cells [124], the role of senescent TECs in the thymic environment should also be explored in the context of positive and negative selection of T-cells. For example, it has yet to be determined whether the interaction of the developing T-cells with SnCs of the thymus play a role in the development of T-cells with auto-reactivity.

Interestingly, the recruitment of T-cell progenitors to the thymus is similar between young and old mice [125]. The reduced T-cell output has been attributed to the defective microenvironment of the thymus and other secondary lymphoid organs [125–127]. Though there is a significant functional decline in thymic activity, the aged thymus still retains a portion of its function [128], which leads us to believe that the therapeutic clearance of SnCs could help to restore thymic function in the elderly. Thymic regeneration strategies so far have largely failed to improve the production of functional of T-cells, in part due to the lack of a systemic approach, because rejuvenating the thymus alone still leaves the secondary lymphoid organs too impaired to support the naïve T-cells being produced [127, 129].

It would be intriguing to replicate these studies with a senolytic combinatorial therapy to see how it changes the outcome. It should be a promising venture, because caloric restriction, a dietary intervention known to reduce cellular senescence [130] and SASP [131, 132], has been shown to delay thymic involution and mitigate thymic adipogenesis [133].

### Spleen

The spleen is a secondary lymphoid organ that acts as a blood filter to remove damaged red blood cells. It plays a crucial role in maintaining the optimal populations of white blood cells and platelets. The spleen can detect pathogenic invaders in the blood and mobilize the immune system to fight against the pathogens [134].

With advancing age, the cellularity and microarchitecture of the spleen changes significantly accompanied by altered localization of various cells [135]. The distinct demarcation of T-cell and B-cell regions within the white pulp becomes obscure with advancing age. Also, an alteration in the organization and function of stromal cells, marginal zone macrophages and marginal metallophilic macrophages can be seen [136]. An accumulation of SnCs with advancing age has been demonstrated to happen in the spleen. This was shown not only by means of elevated expression of *p16^INK4a^* and SASP factors, but also by means of cell accumulation with elevated DNA damage [50, 137]. It has also been shown that the stromal cell populations of the aged spleen, exhibit an upregulated expression of IL-6, a SASP factor, implying that at least a proportion of these cells could be senescent [138].

Age-dependent changes in the splenic microenvironment impair the phagocytic capacity of macrophages in the marginal zone. While the phagocytic capacity of macrophages from the aged spleen seemed to be less efficient *in vivo*, their *in vitro* phagocytic capacity was similar to those from young mice [139]. Interestingly, induction of SnCs accumulation in the spleen after radiation has been shown to impart similar functional impairments to splenic macrophages in mice, and the clearance of such SnCs was able to restore macrophage function [140]. Microenvironment-dependent dysfunction and impaired migration of B-cells can also be seen in the aged spleen [141]. Even B-cells originating from young HSCs in an aged recipient showed signs of dysfunction, providing support to the idea that B-cell dysfunction is mainly attributable to the aged splenic environment [135].

**Figure 4:**
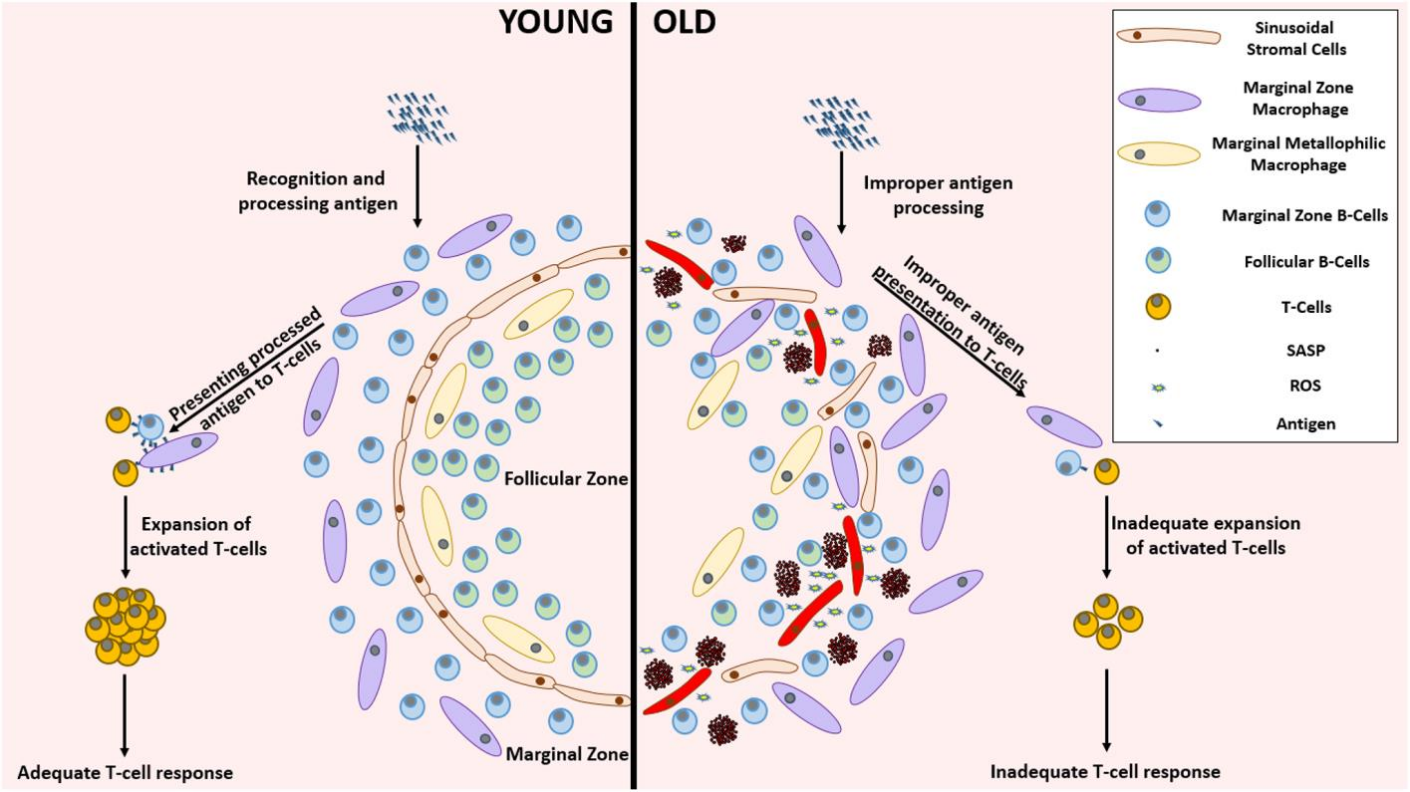
**Remarkable differences between the young and aged splenic environment.** With advancing age, the stromal cells in the lining of sinuses, that demarcate follicular zone from the marginal zone, become less organized accompanied with an altered localization of various cell types. The inflammatory environment created by the accumulation of SnCs impairs the functionality of several cells residing in the spleen. This functional impairment mediated improper antigen presenting capabilities lead to the establishment of an inadequate T-cell response against pathogenic invasion. Abbreviations: SnC: Senescent cell; SASP: Senescence associated secretory phenotype; ROS: Reactive Oxygen Species.

Splenic priming of T-cells is a crucial step in the establishment of an appropriate T-cell response [142]. It is known that the senescent splenic environment impairs the recruitment of T-cells to the spleen. In addition, as depicted in (Figure 4), the microenvironment-mediated impairment of the functionality of antigen-presenting cells such as B-cells, macrophages and dendritic cells (DCs) in the aged spleen may explain why even T-cells originating from young HSCs were dysfunctional and showed a delayed response to stimulation in an aged splenic microenvironment [143, 144].

Though not deeply explored in these studies, it is apparent that the splenic environment of the old mice is not conducive for the proper functionality of various immune cells. Apart from SASP-mediated micro-environmental alterations, SnCs, by virtue of their altered morphology, can imbue structural alterations to the aged spleen. With senolytics [14, 15, 145–148] and senostatics [149] becoming more accessible, further insights into SASP-independent mechanisms of SnCs involvement in immunosenescence should be explored.

### Lymph nodes

Lymph nodes are small bulbous structures that form a crucial part of the lymphatic system along with the lymphatic vessels. They filter the lymph fluid obtained from the surrounding tissues before it re-enters the blood stream [150].

Lymph nodes house various immune cells including T-cells, B-cells and DCs and play an essential role in establishing a strong immune response [151–154]. With advancing age, there is a significant decline in the number, integrity, and functionality of lymph nodes [135, 155–158]. Alterations in cellularity and functionality of different cell types of lymph nodes have been shown to occur with advancing age (reviewed here [158]). Increased adiposity and fibrosis have also been described in lymph nodes of patients older than 60 years [155, 156].

It has been speculated that lymphatic endothelial cells and high endothelial venules of the lymph nodes show signs of aging similar to that of the vascular system. This includes altered permeability, accumulation of SnCs, and increased inflammation, which could act as causal factors that adversely affect the migration and recruitment of immune cells like naïve T-cells [158]. It has also been shown that the age-dependent increase in the level of prostaglandin-2 in the lungs inhibits the migration of DCs to the draining lymph nodes, leading to the establishment of an improper T-cell response to viral infections like SARS-CoV [159]. This is interesting since prostaglandin production is upregulated in SnCs [160], and provides evidence on how cellular senescence in other organs can indirectly impact the function of lymph nodes.

Stromal cells from aged lymph nodes have reduced replicative potential upon stimulation [161, 162] and were unable to support naïve T-cell homeostasis [127]. Though not explored as a possibility in these studies, this could be an indication that at least a portion of these stromal are senescent. Another interesting study sheds light on the role of chemokine ligand 2 (CCL2) produced by the stromal cells of lymph nodes in the mitigation of antibody response [163]. Despite this being an important function that prevents the establishment of unnecessary germinal centers in the absence of an antigen, CCL2 is a SASP factor, which raises the question of whether senescent stromal cells that perpetually produce CCL2 are responsible for the age-dependent impairment of lymph nodes to support germinal centers [157].

It seems highly likely that cellular senescence is involved in this age-related lymph node deterioration. Further studies exploring the presence of SnCs in the aged lymph nodes and their role in lymph node-mediated immune response are needed.

### Mucosa associated lymphoid tissue

Mucosa-associated lymphoid tissue (MALT) is a part of the immune system that localizes on the surface of the mucosal tissues. Depending on their location, MALT is classified into different types, such as inducible bronchus-associated lymphoid tissue (iBALT) [164, 165], conjunctiva-associated lymphoid tissue (CALT) [166, 167], larynx-associated lymphoid tissue (LALT) [168] and inducible skin-associated lymphoid tissue (SALT) [169, 170]. The most commonly studied MALT representatives are nasopharynx-associated lymphoid tissue (NALT) [171, 172] and gut-associated lymphoid tissue (GALT) [173].

In humans, the adenoids of the nasopharynx, tonsils of oropharynx, and a few more lymph nodes in the region form the Waldeyer’s ring [174, 175]. They are considered to be a part of the MALT and are analogous to the NALT in rodents [172]. They are crucial for immunization through intranasal vaccination [176]. Similarly, GALT is comprised of Peyer’s patches, mesenteric lymph nodes (MLNs) and isolated lymphoid follicles (ILFs) [177].

The MALT functions in a complex manner (reviewed here [178]), which is known to be affected by the process of aging, as seen in mice by the age-dependent reduction in the establishment of oral tolerance to novel antigens [179]. This deterioration varies regionally, with NALT conserving its functionality for longer than GALT, making nasal immunizations an attractive alternative for vaccinating the elderly [180, 181].

Though cellular senescence has been shown to be present in the tonsils of patients with tonsillitis and tonsillar hypertrophy, it is still unclear whether SnCs play a role in these pathological conditions [182, 183]. Despite knowing that tonsillar mesenchymal stem cells can undergo cellular senescence [184, 185], the implications of cellular senescence in alterations of the function of NALT has not yet been studied.

Extensive studies in mice show that GALT exhibits a similar age-associated alteration in the cellular composition and decline in functionality like many of the other parts of the immune system. There is a decline in naïve T-cell and B-cell repertoires which are primarily replaced by memory cells [186, 187]. An age-dependent impairment in proliferative response to mitogenic stimulus is also seen in GALT [188]. There is a quantitative decline in dendritic cells accompanied by impaired functionality [189, 190] that yields a similarly impaired priming of T-cells, which is seen in the aged spleen [135, 140, 141]. This impaired immune function, with possible senescence accumulation could explain the age-associated increased rate of cancer incidence in the gastrointestinal tract.

Despite the lack of direct evidence, with the support of pre-existing knowledge of age-associated functional decline and senescence accumulation in organs [191–194] and systemic vasculature [195, 196] associated with these mucosal lymphoid tissues, it is exceedingly convincing that there is an age-dependent accumulation of SnCs in these sites and/or that their functionality is somehow impacted by this accumulation. A speculative supporting argument for this is that the mucosal surfaces are exposed to more environmental stressors than most other organs, which could possibly cause low-grade chronic activation of their immune system and SnCs accumulation. This could explain why we see a relatively early onset in the aging of the mucosal immune system compared to the systemic immune system [180, 181, 186, 197].

Apart from all the circumstantial and correlative evidence, more studies are required to further our understanding of the role of cellular senescence in age-associated changes in MALT and how or if senolytics can rejuvenate them.

## CONCLUSION

As summarized in (Table 1), even at an organ level, the age-associated changes that contribute to immunosenescence are multifaceted with a wide variety of undesirable phenotypic manifestations. Thus, it would be ill-advised to address each of these problems individually. A more feasible and effective way to deal with immunosenescence would be to tackle the fundamental aspects of aging that drive immunosenescence. With studies showing that clearing SnCs can rejuvenate entire tissues and organs of the aged immune system [63, 140], cellular senescence is certainly one such fundamental aspect, which has the potential to address immunosenescence.

**Table 1:** Age-associated changes in the lymphoid organs that contribute to immunosenescence.

**Organ**	**Age-Associated Changes**	**References**
Bone Marrow	↑ Senescent Hematopoietic Stem Cells	[106, 198]
	↑ Senescent Mesenchymal Stem Cells	[95]
	↑ Adiposity	[83, 88, 99]
	↑ Myelopoiesis	[88] [78, 80, 82]
	↓ Lymphopoiesis	[88]
	↑ Oxidative Stress	[95, 96]
	↑ DNA damage	[63, 94, 95, 108, 199]
	↑ Inflammation	[95, 102]
	↓ HSC functionality	[63, 68, 77, 200]
Thymus	↓ Structural Integrity	[111, 112]
	↑ Senescent Thymic Epithelial Cells	[121]
	↑ Adipocytes	[112]
	↑ Fibrosis	[129, 201]
	↑ Inflammation	[122]
	↑ DNA damage	[121]
	↑ Oxidative Stress	[121]
	↓ Naïve T-cell turnover	[125, 126]
Spleen	↓ Structural Integrity	[135]
	↓ Macrophage Phagocytosis	[139]
	↑ Cellular Senescence	[50, 137]
	↓ Migration of B-cells	[135, 141]
	↓ Antigen Presenting Functionality	[135, 144]
	↓ Recruitment of T-cells	[143]
Lymph Nodes	↓ Number	[135, 155]
	↓ Structural Integrity	[135, 156]
	↓ Functionality	[158, 162, 202]
	↑ Adiposity	[155, 156, 158]
	↑ Fibrosis	[155, 156, 158]
Mucosa Associated Lymphoid Tissue	↓ Naïve B-cell repertoire	[186]
	↓ Naïve T-cell repertoire	[186]
	↑ Memory B-cells	[186]
	↑ Memory T-cells	[186]
	↓ Functionality	[188, 189]
	↓ Dendritic Cell Number	[190]
	↓ Dendritic Cell Functionality	[189, 190]

Cellular senescence, because of its involvement in several age-related dysfunctions and disorders, has become an essential area of interest in the field of aging research. Despite a great deal of assimilated knowledge on this phenomenon, there still remain unanswered questions. The role of cellular senescence in immunosenescence is one such key area needing further exploration. With few publications addressing the direct involvement of cellular senescence in specified immunological contexts, and many more studies providing evidence for a possible role of cellular senescence in impeding the function of the immune system, this is an area of research that deserves further exploration and an investment of resources.

In this proposed pursuit, there are several “low-hanging fruit”. A few such addressable questions include: Do SnCs play a direct or indirect role in age-related disparities seen in inflammatory pathological conditions like sepsis? Does SnCs accumulation in the peripheral tissues of the body impact the functionality of immune cells in the central nervous system? Can clearing SnCs hinder the pace of thymic involution? Can clearing SnCs in combination with thymic rejuvenation therapies in the elderly improve thymic function? Does cellular senescence drive age-associated autoimmunity? Can clearing SnCs or inhibiting SASP boost the functionality of different immune cells? Does cellular senescence play a direct role in the impaired vaccination efficacy in the elderly? Is there a senostatic/senolytic regimen that can be followed before and after vaccination to boost its efficacy in the elderly?

The increasing array of genetic models of SnC clearance along with a growing panel of senolytic and senostatic agents, provide a unique opportunity for scientists to answer these questions to lay a strong foundation to this new avenue of research in immunosenescence. Ultimately, gaining a deeper understanding of the interaction between cellular senescence and immunosenescence will help in the development of improved therapeutics that will aid in the conservation of our vitality as we age.
